# Systematic Review and Meta-Analysis of the Efficacy and Safety of Metformin and GLP-1 Analogues in Children and Adolescents with Diabetes Mellitus Type 2

**DOI:** 10.3390/children9101572

**Published:** 2022-10-18

**Authors:** Elisabeth Carydias, Andoneta Tasho, Chara Kani, Flora Bacopoulou, Charikleia Stefanaki, Sophia L. Markantonis

**Affiliations:** 1 Department of Pharmacy, School of Health Sciences, National and Kapodistrian University of Athens, 15774 Athens, Greece; 2 University Research Institute for the Study of Genetic and Malignant Disorders in Childhood, Medical School, National and Kapodistrian University of Athens, 11527 Athens, Greece; 3 Center for Adolescent Medicine and UNESCO Chair in Adolescent Health Care, First Department of Pediatrics, Medical School, National and Kapodistrian University of Athens, 11527 Athens, Greece

**Keywords:** diabetes, insulin, GLP-1 analogues, adolescents, children, metformin, exenatide, liraglutide

## Abstract

Diabetes mellitus type 2 (DMT2) is one of the most frequent glucose metabolism disorders, in which serum glucose concentrations are increased. In most cases, changes in lifestyle and diet are considered as the first step in addressing its therapy. If changes in lifestyle and diet fail, drugs, such as metformin, must be added. Lately, apart from metformin or insulin, the FDA has approved the use of glucagon-like peptide-1 (GLP-1) analogues for children and adolescents. Little is known about their efficacy and safety at this young age. The main aim of this systematic review/meta-analysis was to assess the safety and efficacy of metformin and GLP-1 analogues, exenatide and liraglutide, compared with placebos or other antidiabetic drugs used for DMT2 in children and adolescents. Metformin did not seem to demonstrate pharmacologic superiority, while GLP-1 analogues were found superior to placebos. GLP-1 analogues may be considered a useful alternative for the treatment of DMT2 in children and adolescents.

## 1. Introduction

Diabetes mellitus type 2 (DMT2) is characterized by an impairment in the way the body regulates and uses glucose as a fuel. It is a chronic condition resulting in increased glucose circulating in the bloodstream, which eventually can lead to disorders of the circulatory, nervous, and immune systems. DMT2 has received attention during the last 30 years due to its rise in all age groups, even in children and adolescents, altering its epidemiologic trend from a rise in 1990 to an epidemic in the last decade [1,2]. Obesity epidemiologic markers, especially in children, have also dramatically increased over the last few decades [3,4]. It seems that the median age of onset for DMT2 is 13.5 years of life, while its prevalence rises during the second decade of life [5,6]. The onset of DMT2 in the young is associated with the physiologic phenomenon of insulin resistance during adolescence, as well as with genetic and epigenetic cues [7,8,9,10]. Risk factors, such as family history of diabetes, obesity, metabolic syndrome, lack of exercise, sedentary lifestyle, and socio-economic status are implicated in the current rise in the prevalence of DMT2.

Current therapeutic choices for DMT2 in adults are, first, changes in lifestyle and, in case of failure, the following drugs: metformin, sodium-glucose cotransporter-2 inhibitors (SGLT2i), glucagon-like peptide-1 (GLP-1) analogues, dipeptidyl peptidase (DPP-4 inhibitors), thiazolidinediones (TZDs), sulfonylureas-(SU), and insulin. Other categories of pharmacologic interventions are meglitinides (GLN), alpha-glucosidase inhibitors (AGI), colesevelam, amylin analogues, and agonists of the dopamine D2 receptor. The choice of the pharmacologic intervention is based on the following parameters: efficacy, safety, adverse reactions, mechanism of action, risk of hypoglycemia, impact on weight gain or weight loss, efficacy on various physiologic systems such as the cardiovascular system and the kidneys, patient-friendly process, and the economic cost.

It has been estimated that there were approximately 41,600 new cases of diagnosed DMT2 among children and adolescents in 2021 worldwide. Around 30% and 40% of the worldwide total incident cases were in the IDF Western Pacific region and in World Bank upper-middle-income countries, respectively. The three countries with the highest estimated number of incident cases were China, India, and United States of America [11]. Therefore, the need for new pharmaceutical agents in the pediatric/adolescent populations is indispensable. The FDA approved the use of liraglutide, a GLP-1 analogue, in children and adolescents with DMT2 in 2019 [12]. The same kind of approval was given for metformin in 2000 and for exenatide in 2021. Children and adolescents with premature pubarche and polycystic ovary syndrome have a considerable degree of insulin resistance. The insulin-sensitizing actions of metformin encouraged many investigators and physicians to use it as the key drug in these conditions for both prevention and treatment. However, long-term controlled studies are still required to assess the degree and duration of the effectiveness and safety of metformin [13]. The aim of this systematic review and meta-analysis was to summarize the current medical literature on the efficacy and safety of metformin and GLP-1 analogues used for DMT2 in children and adolescents.

## 2. Materials and Methods

### 2.1. Eligibility Criteria

Randomized controlled trials (RCTs) of male or female children and adolescents, 8–21 years of age with DMT2, assessing metformin or GLP-1 analogues as monotherapy or as an add-on therapy vs. placebo or any other antidiabetic drug, were included in the meta-analysis.

More specifically, eligible studies were any RCTs that assessed metformin or GLP-1 analogue vs. placebo; metformin or GLP-1 analogue vs. any other antidiabetic drug; metformin or GLP-1 analogue as add-ons vs. placebo; metformin or GLP-1 analogue in combination with other antidiabetic drugs vs. placebo.

### 2.2. Information Sources

The PubMed, the Cochrane Library, the EMBASE, and the Clinical Trials databases were used as sources up to 31 March 2022. This meta-analysis has been registered in PROSPERO and has received a registration number (CRD42022312855).

### 2.3. Search Strategy, Selection Process, Data Collection Process

A search strategy was developed by the independent reviewers. The algorithmic procedure resulted from the combination of the following MESH terms: diabetes OR diabetes mellitus AND children OR adolescents OR pediatric use OR metformin OR GLP-1 analogues. Restrictions of human species and randomized controlled trials were applied. Reference lists of relevant articles were hand searched for potentially eligible studies to maximize the amount of evidence.

### 2.4. Data Items, Outcome Assessment

The efficacy of metformin and GLP-1 analogues was assessed by hemoglobin A1c (HbA1c), fasting plasma glucose concentrations (FPG), body mass index (BMI), and body weight (BW). Assessment of the safety of the metformin and GLP-1 analogues was performed by the reporting of adverse events.

### 2.5. Study Risk of Bias Assessment

The quality of the studies included in this meta-analysis was assessed via the risk of bias tool of the Cochrane handbook, using the RevMan program. Random allocation generation, allocation concealment, blinding of participants and personnel, blinding of outcome assessment, incomplete outcome data, selective reporting, and other biases were used as parameters for risk bias assessment. Risk bias was assessed for every single study and was characterized as low, medium, or high.

### 2.6. Synthesis Methods

The RevMan 5 program was used for this meta-analysis. The minimum number of studies included for meta-analysis is 2. The random-effect model was used due to different interventions in the studies included in this meta-analysis. HbA1c, FPG, BMI, and BW were assessed via standardized mean difference (SMD) and confidence interval of 95% (adjusted and non-adjusted mean, least square mean). Mean values with standard deviation, and standard error were inserted or calculated, using the calculator of RevMan (estimated treatment difference tool). Odds ratio (OR) and the confidence interval of 95% were used regarding the dichotomous variables of adverse events, accompanied by the numbers of patients that demonstrated adverse events to the total number of participants in every study. Heterogeneity was assessed with the use of Ι^2^ and *p*, where *p* < 0.1 or Ι^2^ > 50% were considered as high.

### 2.7. Certainty Assessment

GRADE (grading of recommendations assessment, development, and evaluation) was employed to assess the level of certainty of the results of the studies [14].

## 3. Results

### 3.1. Study Selection

**Figure 1:**
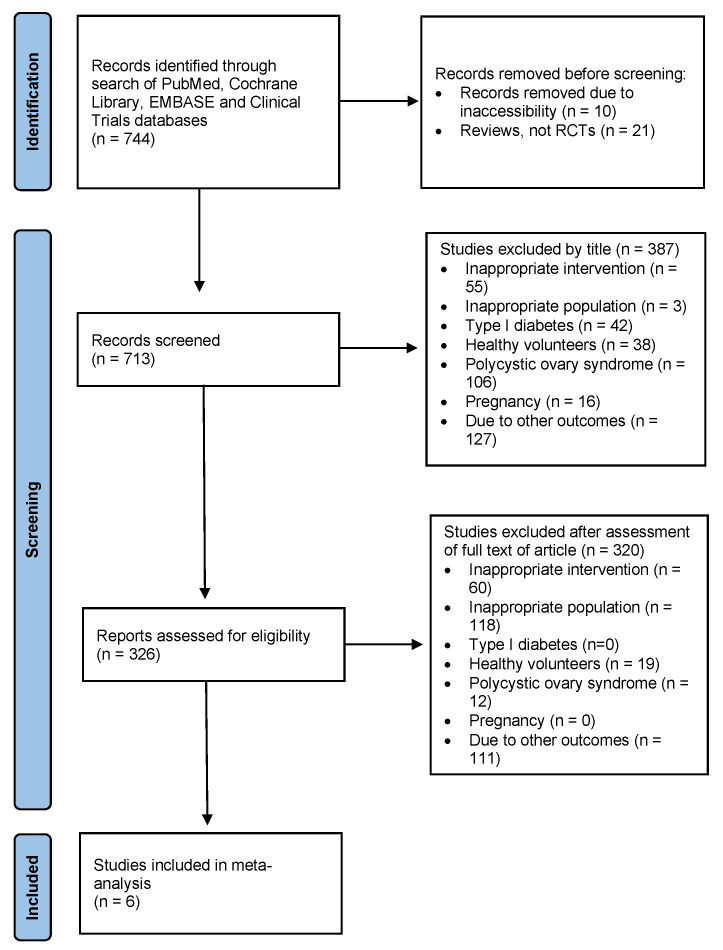
PRISMA flow diagram displaying results of the literature search.

The search strategy resulted in 744 papers, of which 10 papers were impossible to acquire; 21 papers were systematic or narrative reviews; 105 papers assessed other drugs; 42 papers assessed diabetes mellitus type 1; 19 papers assessed healthy controls; 118 papers assessed polycystic ovary syndrome (PCOS); 238 papers assessed different topics; 16 papers assessed gestational diabetes. Finally, only six papers were included in this meta-analysis (Figure 1).

### 3.2. Study Characteristics

Three randomized controlled trials were assessed for the metformin comparisons: the Today Study Group, 2013 trial (quadruple-blind, 699 participants) [15], the Jones et al., 2002 trial (double-blind, 82 participants) [16], and the Gottschalk et al., 2007 trial (single-blind, 284 participants) [17]. In addition, three randomized controlled trials were assessed for the GLP-1 analogues’ comparisons: the Klein et al., 2014 trial (double-blind, 19 participants) [18], the Tamborlane et al., 2019 trial (double-blind, 134 participants) [19], and the National Library of Medicine NCT00658021 trial (double-blind, 122 participants) [20].

### 3.3. Results of Syntheses

#### 3.3.1. Metformin

**Figure 2:**
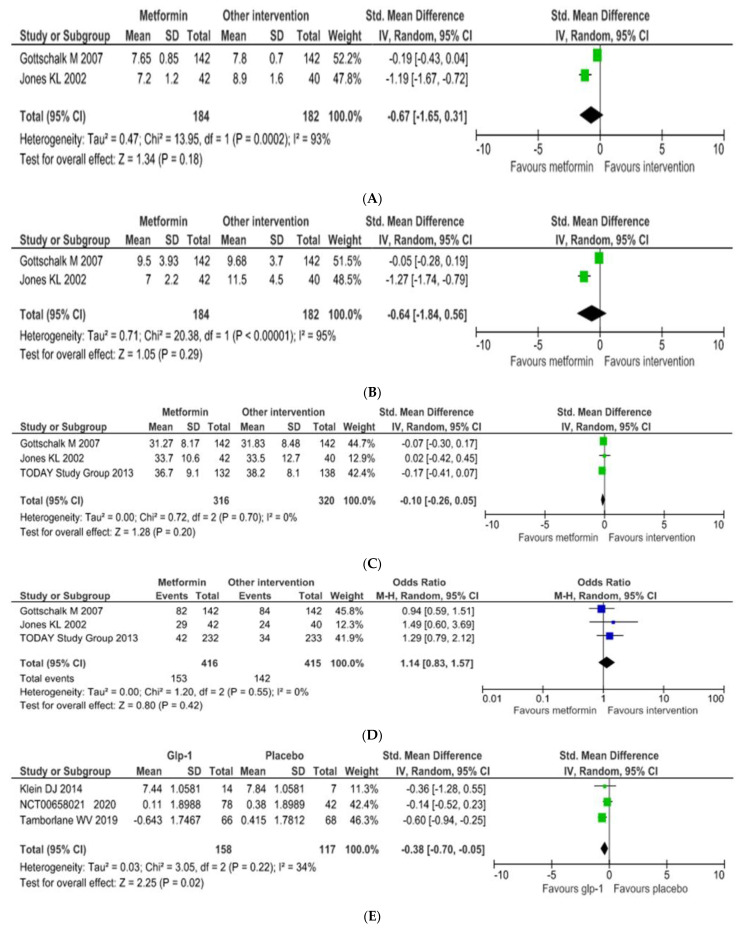

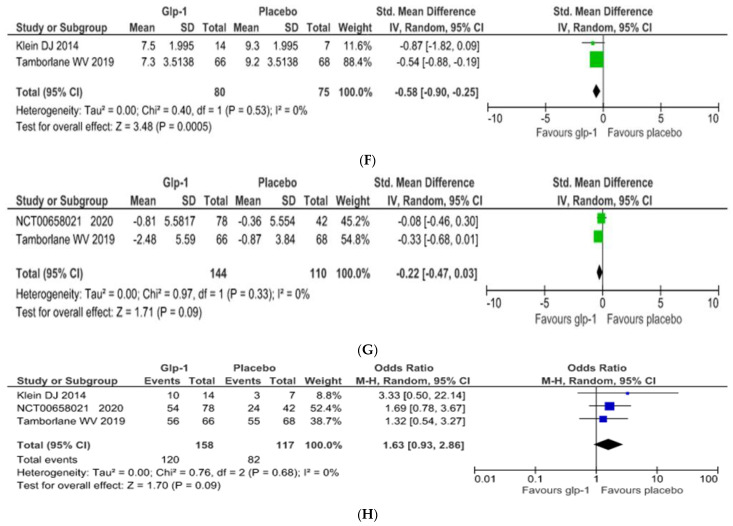
Forest plot comparisons of metformin or liraglutide or exenatide versus placebo, glimepiride, and rosiglitazone. (**A**) Outcome: HbA1c values (**B**) Outcome: fasting plasma glucose (**C**) Outcome: BMI (**D**) Outcome: adverse events (**E**) Outcome: HbA1c values (**F**) Outcome: fasting plasma glucose (**G**) Outcome: BMI (**H**) Outcome: adverse events.

Based on the HbA1c values from the included studies it appears that metformin did not lead to significantly better outcomes than glimepiride, placebo, or rosiglitazone (SMD equal to −0.67 [95% CI: −1.65–0.31] and I^2^ = 93%) (*p* = 0.18) (Figure 2A). Fasting plasma glucose demonstrated the same trend with HbA1c. SMD was equal to −0.64 ([95% CI: −1.84–0.56] and I^2^ = 95%) (*p* = 0.29) (Figure 2B). Based on BMI values, metformin did not demonstrate better outcomes than the other interventions (*p* = 0.20). SMD was equal to −0.10 (95% CI: [−0.26–0.05], and I^2^ = 0%) (Figure 2C). Finally, the incidence of adverse events for the metformin groups was higher (OR = 1.14; [95% CI: 0.83–1.570]), but not to a significant extent (*p* = 0.42) (Figure 2D). There was no observed heterogeneity in the number of events noted in the studies.

#### 3.3.2. GLP-1 Analogues

All three double-blind RCTs compared a GLP-1 analogue with a placebo. HbA1c values were significantly lower in the GLP-1 analogues groups when compared with the placebo in all three studies, with only moderate heterogeneity (SMD = −0.38; 95% CI: −0.70–−0.05; I^2^ = 34%) (*p* = 0.02) (Figure 2E). Fasting plasma glucose values followed the same trend as the HbA1c values, i.e., favoring liraglutide and exenatide, with no observed heterogeneity (SMD = −0.58 [95% CI: −0.90–−0.25]; I^2^ = 0%) (*p* = 0.0005) (Figure 2F). GLP-1 analogues did not seem to have a greater effect than the other interventions on weight loss (SMD = −0.22 [95% C.I: −0.47–0.03]; I^2^ = 0, (*p* = 0.09) (Figure 2G). Finally, as with metformin, the incidence of adverse effects was higher with the GLP-1 analogues than the placebo, but the difference was not statistically significant (SMD = 1.63 [95% CI: 0.93–2.86]; I^2^ = 0%) (*p* = 0.09) (Figure 2H).

### 3.4. Reporting Biases

**Figure 3:**
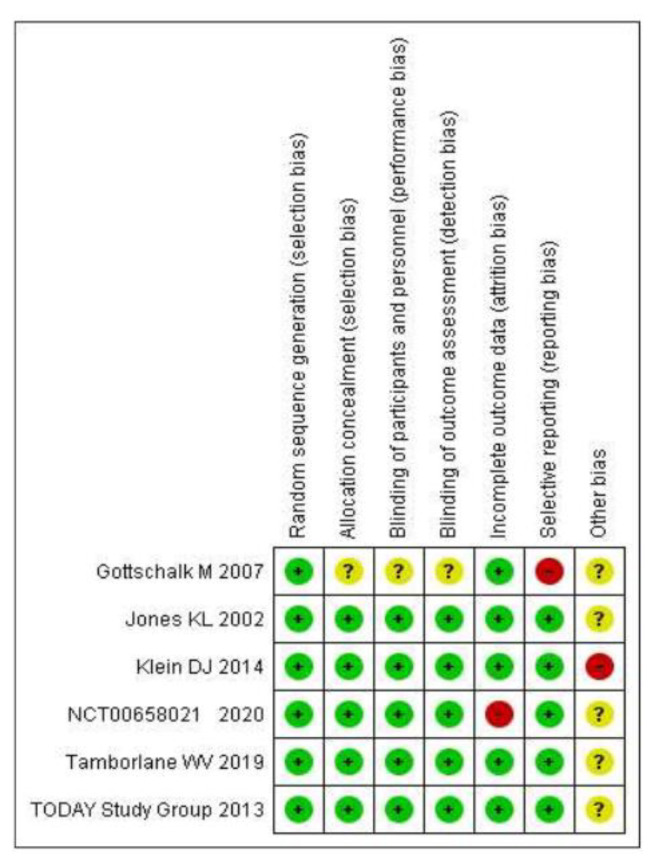
Summary of the bias report. The green color indicates low risk of bias, yellow color indicates unclear risk of bias and red color indicates high risk of bias.

Regarding random sequence generation, the risk of bias in all included studies was considered low. Regarding allocation concealment, the risk was considered unclear in the study of Gottschalk et al., 2007 [17] and low in the others. In addition, for the latter study, for the blinding of participants and personnel and the blinding of outcome assessments, the risk was considered unclear due to it being a single-blind trial, while for the other studies it was considered low. Lastly for the Gottschalk et al., 2007 study [17], the risk of selective reporting bias was assessed as high, as the exact values are not given for some indicators, but for the other five studies it was considered low. Regarding incomplete outcome data, the risk was high in the NCT00658021 study [20], as the proportion of patients who discontinued treatment with each intervention was different. In contrast, this risk in the other studies was low. Finally, the risk of other bias was unclear because there was insufficient information to document it. Only in the Klein et al., 2014 study [18], the risk was considered high because the small number of participants and the short duration of treatment cast doubt on drawing reliable conclusions. Figure 3 summarizes the bias report.

### 3.5. Assessment of Level of Certainty of Results (GRADE)

The GRADE approach provides a structured way to consider the following factors: risk of bias [21,22], precision of effect estimates [23], consistency of individual study results [24], how directly the evidence answers the question of interest, and risk of publication or reporting biases [25,26] to assess the certainty of evidence for each outcome in a meta-analysis. The certainty in evidence or quality of evidence is graded as: very low, low, moderate, and high.

As all studies in this meta-analysis were RCTs, they were initially graded as high and subsequently, depending on the severity of each factor, the rating was reduced by one (1) or two (2) levels. The grades were not lowered as a result of publication bias or indirectness, implementing the PICO (population, intervention, comparison, outcome) format, as the risk was considered low. For all comparisons involving metformin, the grade was lowered by one step due to risk of bias, because the study with the highest percentage weight in all comparisons was a single-blind trial with an unclear risk of allocation concealment and blinding while having a high risk of selective reporting bias. More specifically:

*For the direct comparison of the outcomes for Metformin vs. other interventions:*

The grade was estimated to be very low for the glycated hemoglobin values. This was mainly due to the high heterogeneity (inconsistency) based on an I^2^ value of 93% (*p* = 0.0002), so the quality of evidence was downgraded by two steps The imprecision of the values was considered high due to the wide range of the CI around the best estimate of the absolute effect [−1.65–0.31], but not enough to downgrade one more point, since the number of participants was greater than 300 (366).

For the fasting plasma glucose outcome, the grade was very low due to high heterogeneity (I^2^ = 95%, *p* < 0.00001) (resulting in a twostep downgrade) and high imprecision as the overall CI was [−1.8–0.56] with 366 participants (one step downgrade).

For the body mass index, the grade was estimated to be moderate due to the risk of bias. The heterogeneity (I^2^ = 0%, *p* = 0.70) and imprecision (CI [−0.26–0.05] with 636 participants) were low, so the grade was not downgraded further.

Finally, regarding the incidence of adverse effects, the grade was estimated to be moderate due to the risk of bias, as heterogeneity (I^2^ = 0%, *p* = 0.55) and imprecision (CI [0.83–1.57] with the number of adverse events equal to 295) were low.

*For the direct comparisons of the outcomes for the GLP-1 analogues vs. placebo:*

The grade was estimated to be low for the glycated hemoglobin values due to a high risk of bias, as one of the three studies with the greatest percentage weight had incomplete outcome data and other source of bias (one step downgrade). Heterogeneity was not considered important (I^2^ = 34%, *p* = 0.22), but, due to imprecision (CI [−0.70, −0.05] with 275 participants), a one-step downgrade was made.

For fasting plasma glucose, the grade was estimated to be moderate due to high imprecision (CI [−0.90–−0.25] with 155 participants) and thus there was a one-step downgrade. Heterogeneity was low (I^2^ = 0%, *p* = 0.53) as was the risk of bias, as the study with the greatest effect had an unclear risk of other source of bias.

Regarding the BMI values, the grade was estimated to be low due to a high level of imprecision (CI [−0.47–0.03] with 254 participants) and a high risk of bias, as both studies with almost the same percentage weight had incomplete outcome data on the one hand and an unclear risk of other bias on the other. Heterogeneity was low (I^2^ = 0%, *p* = 0.33), so no further downgrade was made.

Finally, regarding the incidence of adverse effects, the grade was estimated to be low due to high imprecision (CI [0.93–2.86], number of events = 202) and a high risk of bias, as one of the three studies with a high percentage weight had incomplete outcome data and the study with the highest percentage weight had an unclear risk of other source of bias. This resulted in a one-step lower grade. Heterogeneity was low (I^2^ = 0%, *p* = 0.68).

## 4. Discussion

DMT2 is traditionally acknowledged as a disease of older age and it is considered as relatively rare in pediatric and adolescent populations. However, it has been showcased in several studies in parallel with rising rates of childhood obesity and has been on the rise in pediatric and adolescent patients over the past two decades in many countries of the modern westernized societies. Therefore, the need for newer pharmaceutical agents seems to be imperative [11]. The main aim of this meta-analysis was to assess the efficacy and safety of metformin and GLP-1 analogues in children and adolescents with DMT2. The comparison of these drugs with other antidiabetic interventions can assist clinicians in making appropriate clinical decisions. The primary outcomes were the values of HbA1c, FPG, BMI, and BW. The results of the meta-analysis suggest that the efficacy of metformin is not statistically greater than that of glimepiride, a placebo, or the combination of metformin and rosiglitazone. The GLP-1 analogues (liraglutide and exenatide) seem to be more effective than a placebo in reducing HbA1c and FPG. Safety was assessed by the presence of adverse events. The risk of adverse events seems to be greater for metformin and the GLP-1 analogues than the other interventions, but the results of the comparisons were not statistically significant. Therefore, these antidiabetic drugs do not appear to be safer in children and adolescents with DMT2.

Our results are consistent with those of Al Shareef et al., [27], who demonstrated that HbA1c, FPG, ΒW, and lipid profile were marginally better in the metformin groups, and concluded that there was limited but not convincing evidence to suggest that metformin can improve the glycemic control in children and adolescents with DMT2 compared with other interventions. In addition, there were more adverse events in the metformin groups, but these were not statistically significant. Metformin seems to be safe and effective for treatment of DMT2 in pediatric patients [28]. Plus, one significant meta-analysis concluded that the administration of metformin in children and adolescents resulted in a dose–response relationship between metformin use and increases in height in those populations compared with controls. While an approximate 1-cm increase in height may appear small, it seemed likely underestimated given that many studies were of short duration and included older adolescents, potentially after epiphyseal growth plate closure [29].

A recent meta-analysis about the efficacy of GLP-1 agonists in obesity and DMT2 concluded that GLP-1 agonists demonstrate modest effects on the glycemic control and weight loss; the authors attributed those findings to the low drug dosage [30]. In addition, therapy of DMT2 with GLP-1 agonists seemed to be well tolerated in pediatric populations, with only mild side effects and no severe episodes of hypoglycemia. Gastrointestinal symptoms seemed to be most frequent, as those in other studies of adult patients [31,32], and consistent with the known effects of GLP-1 agonists on delaying gastric emptying [33]. Chadda et al., concluded that these findings are indicative of detectable glycemic benefits of GLP-1 agonists in children with obesity without diabetes, as well as in those with prediabetes or established DMT2 [30]. Monami et al., [34] performed a meta-analysis in 2009. They assessed the efficacy of liraglutide and exenatide vs. placebo, glimepiride, and insulin in adults with DMT2. HbA1c and BMI were decreased in the GLP-1 analogues groups, while the low risk of hypoglycemia and other adverse effects corroborated the safety of these drugs. However, in this meta-analysis, a larger number of studies were included, the intervention was performed in adults, and the comparators were placebo, glimepiride and insulin.

Several limitations have to be acknowledged. As far as GLP-1 analogues are concerned, only liraglutide and exenatide were evaluated. GLP-1 analogues have to be evaluated as a group for their efficacy and safety in children and adolescents with DMT2. Another limitation is the pharmaceutical financing of a number of these studies, that may have compromised their validity by introducing bias in the safety and efficacy outcomes of these drugs. In addition, the meta-analysis included a small number of studies and a limited and varied number of participants adhering to and discontinuing each intervention, thus affecting the quality of evidence.

## 5. Conclusions

The repertoire of drugs available to treat DMT2 in children and adolescents is limited [35]. Antidiabetic therapies must be tolerable to facilitate compliance and subsequent efficacy [36]. Metformin does not seem to be more efficacious with respect to its antidiabetic activity than glimepiride, a placebo, or the combination of metformin and rosiglitazone in children and adolescents with DMT2, while GLP-1 analogues seem to be more effective than a placebo. The risk of adverse events seems to be greater for both metformin and GLP-1 analogues, mostly mild adverse gastrointestinal events. Incretin-based therapeutic modalities should be integrated in the guidelines of acknowledged societies about the therapeutic means of DMT2 in children and adolescents, as GLP-1 analogues appear to be a useful therapeutic alternative in children and adolescents with DMT2, but larger well-designed studies need to be performed to confirm our results.

## Data Availability

Not applicable.
